# Statistical Predictions for the Dynamics of a Low-Speed System: Newtonian versus Special-Relativistic Mechanics

**DOI:** 10.1371/journal.pone.0036430

**Published:** 2012-05-11

**Authors:** Shiuan-Ni Liang, Boon Leong Lan

**Affiliations:** School of Science, Monash University, Bandar Sunway, Selangor, Malaysia; University of Nottingham, United Kingdom

## Abstract

The Newtonian and special-relativistic statistical predictions for the mean, standard deviation and probability density function of the position and momentum are compared for the periodically-delta-kicked particle at low speed. Contrary to expectation, we find that the statistical predictions, which are calculated from the same parameters and initial Gaussian ensemble of trajectories, do not always agree if the initial ensemble is sufficiently well-localized in phase space. Moreover, the breakdown of agreement is very fast if the trajectories in the ensemble are chaotic, but very slow if the trajectories in the ensemble are non-chaotic. The breakdown of agreement implies that special-relativistic mechanics must be used, instead of the standard practice of using Newtonian mechanics, to correctly calculate the statistical predictions for the dynamics of a low-speed system.

## Introduction

If the speed of a system is low, that is, much less than the speed of light, it is expected [1]–[3] that the dynamics predicted by special-relativistic mechanics is always well-approximated by the dynamics predicted by Newtonian mechanics for the same parameters and initial conditions. In a recent paper [4], we compared the Newtonian and special-relativistic predicted trajectories for a model Hamiltonian system – the periodically-delta-kicked particle. We found, contrary to expectation, that although the particle speed is low, the Newtonian trajectory does not remain close to the special-relativistic trajectory – the two trajectories eventually become completely different regardless of whether the trajectories are chaotic or non-chaotic. However, the agreement between the Newtonian and special-relativistic trajectories breaks down much faster in the chaotic case compared to the non-chaotic case. Similar breakdown of agreement was also found in a model dissipative system [5], [6] and a model scattering system [7]. The loss of agreement means [6]–[8] that special-relativistic mechanics must be used, instead of the standard practice of using Newtonian mechanics, to correctly calculate the trajectory of a low-speed system.

In this paper, we extend the Newtonian special-relativistic comparison for the low-speed periodically-delta-kicked particle from *single-trajectory* predictions [4] to *statistical* predictions **–** in particular, the mean, standard deviation and probability density function of the position and momentum **–** which are calculated from the same parameters and initial ensemble of trajectories. Calculating these statistical quantities directly from an ensemble of trajectories is far easier than solving the Newtonian and special-relativistic Liouville’s equations numerically to first obtain the phase-space probability density functions. Details of the model Hamiltonian system and calculation are given next, followed by the results and discussion.

## Methods

The model Hamiltonian system is a one-dimensional system where the particle is subjected to a sinusoidal potential which is periodically turned on for an instant. The Newtonian equations of motion for the periodically-delta-kicked particle are easily integrated exactly [9], [10] to yield a mapping, which is known as the standard map, of the dimensionless scaled position *X* and dimensionless scaled momentum *P* from just before the *n*th kick to just before the (*n*+1)th kick:
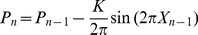
(1)

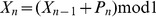
(2)where *n* = 1,2,…, and *K* is a dimensionless positive parameter. For the standard map, the transition from weak (local) chaos to strong (global) chaos occurs at *K*≈0.917.

The special-relativistic equations of motion are also easily integrated exactly, producing a mapping known as the relativistic standard map [11], [12] for the dimensionless scaled position *X* and dimensionless scaled momentum *P* from just before the *n*th kick to just before the (*n*+1)th kick:
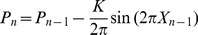
(3)

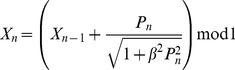
(4)where *n = *1,2, …, and *β*, like *K*, is a dimensionless positive parameter. Since
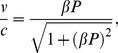
(5)βP<<1 implies v<<c (i.e., low speed), where v is the particle speed and c is the speed of light. Ciubotariu et al. [13] have studied a dissipative version of the relativistic standard map to see how weak damping changes the phase-space structure around the origin described by the relativistic standard map; they did not however compare the dynamical predictions of their dissipative relativistic standard map with the predictions of the dissipative non-relativistic standard map.

For both theories, the ensemble of trajectories is initially Gaussian distributed in position and momentum with means <*X*_0_> and <*P*_0_>, and standard deviations *σ_X0_* and *σ_P0_*:
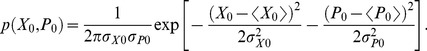
(6)


Each trajectory in the Newtonian (special-relativistic) ensemble is time-evolved using the standard map (relativistic standard map). For each theory, the mean trajectory, i.e., mean position and mean momentum, just before each kick is calculated from the ensemble of trajectories. First, the mean trajectory is calculated using 10^6^ trajectories, where the accuracy of the double-precision calculation is determined by comparison with the quadruple-precision calculation. The mean trajectory is then recalculated using 10^7^ trajectories with the same accuracy determination. Finally, the accuracy of the mean trajectory is determined by comparing the 10^6^-trajectories calculation with the 10^7^-trajectories calculation. The position and momentum standard deviations and probability density functions are calculated in the same manner.

## Results

**Figure 1:**
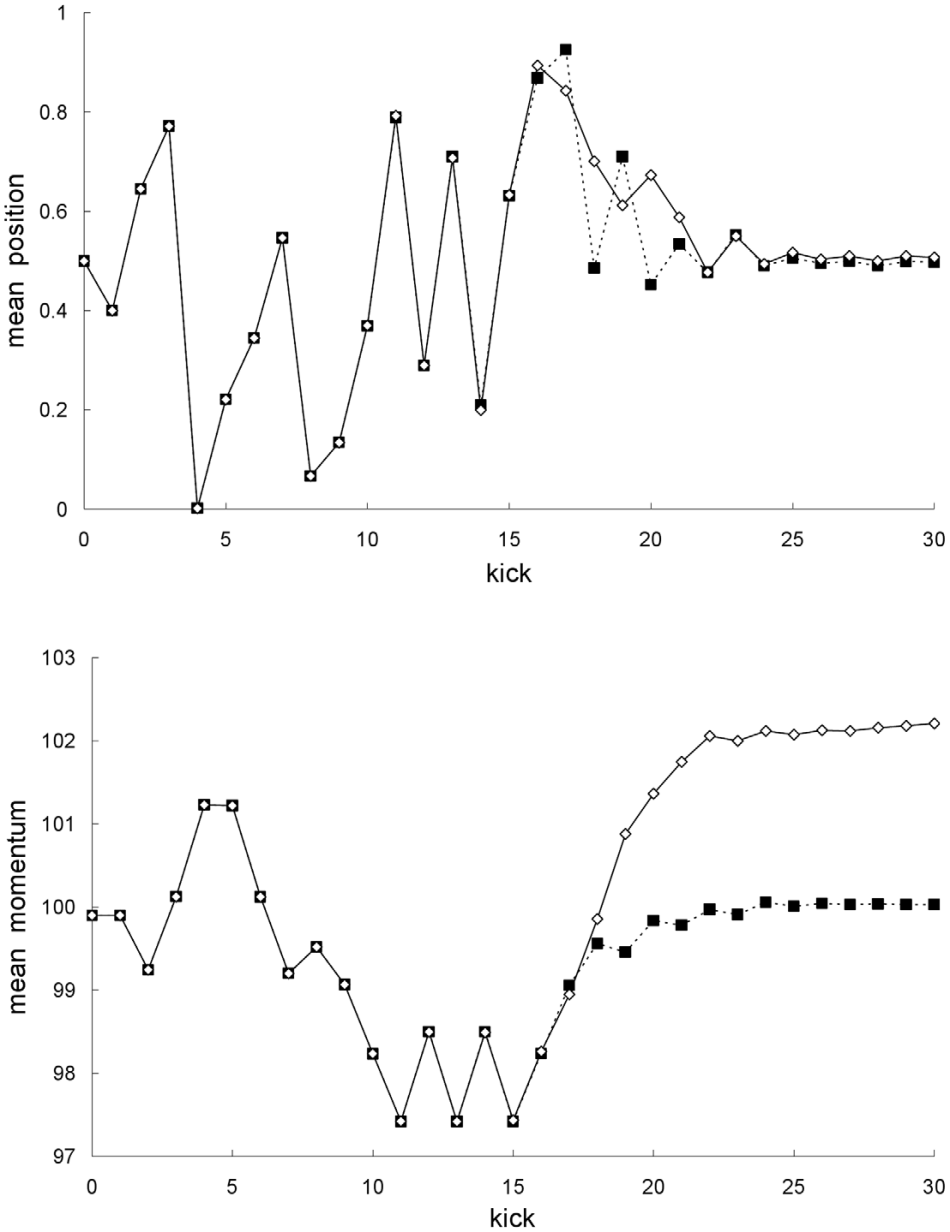
Comparison of mean trajectories for the first example. Newtonian (squares) and special-relativistic (diamonds) mean positions (top plot) and mean momentums (bottom plot) for the first example.

In this section, we will present three examples to illustrate the general results. In the first example, the map parameters are *K* = 7.0 and *β* = 10^−7^. The Newtonian and special-relativistic ensembles are both initially Gaussian distributed in phase space with means <*X*_0_> = 0.5 and <*P*_0_> = 99.9, and standard deviations *σ_X0_* = *σ_P0_* = 10^−10^, and thus initially localized in the chaotic ‘sea’ in phase space. Figure 1 shows that the Newtonian mean position and mean momentum agree with the special-relativistic mean position and mean momentum for the first 16 kicks only, the two mean trajectories are completely different from kick 17 onwards.

**Figure 2:**
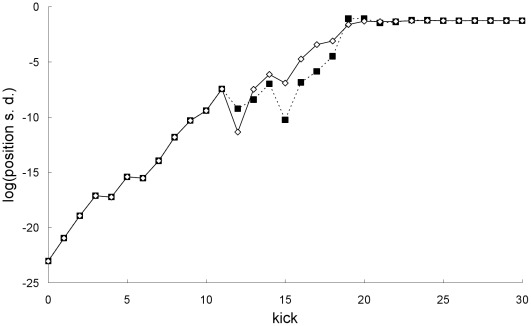
Comparison of position standard deviations for the first example. Natural-log of the Newtonian (squares) and special-relativistic (diamonds) position standard deviations for the first example.

**Figure 3:**
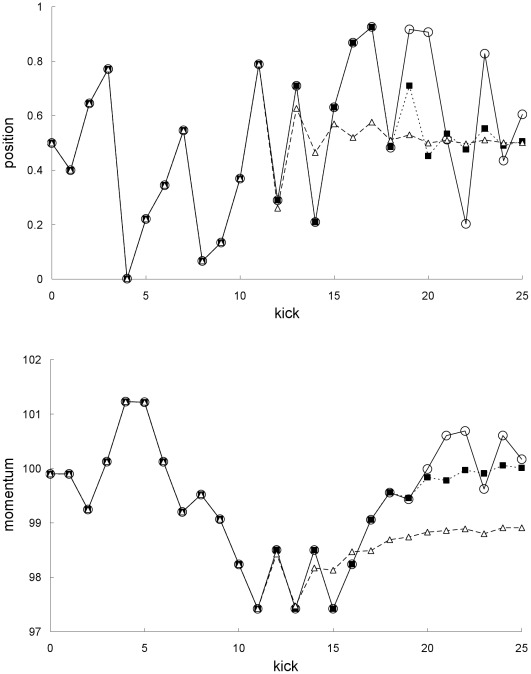
Newtonian single and mean trajectories. Newtonian single trajectory (circles), Newtonian mean trajectory for the first example (squares), and Newtonian mean trajectory for the second example (triangles): positions (top plot) and momentums (bottom plot).

**Figure 4:**
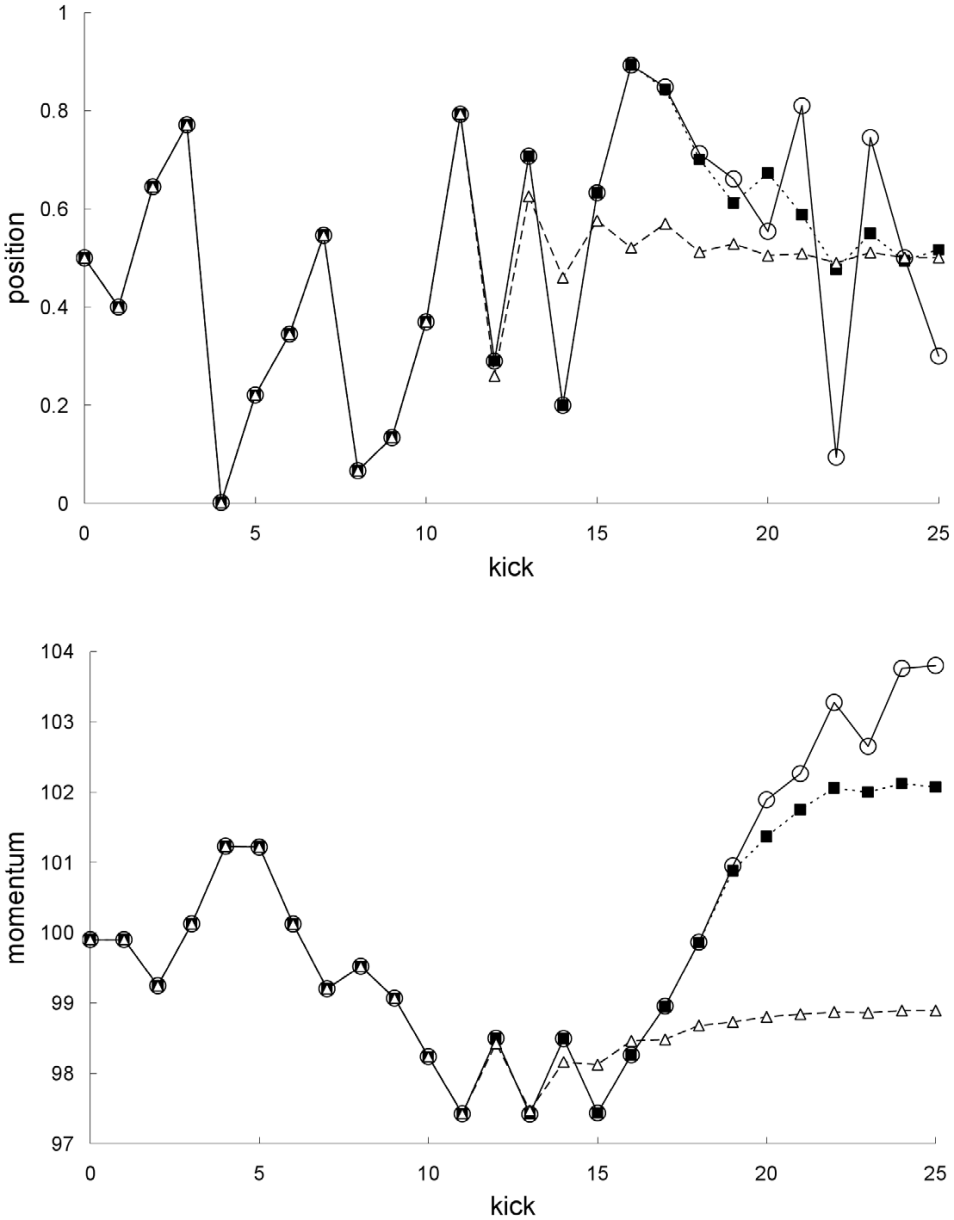
Special-relativistic single and mean trajectories. Special-relativistic single trajectory (circles), special-relativistic mean trajectory for the first example (squares), and special-relativistic mean trajectory for the second example (triangles): positions (top plot) and momentums (bottom plot).

The breakdown of agreement between the Newtonian and special-relativistic mean trajectories in Figure 1 can be understood as follows. In either the Newtonian or special-relativistic case, the position and momentum standard deviations grow [14], [15] exponentially initially because the trajectories in the ensemble are chaotic. But as long as the position standard deviation remains small (<<1), the mean trajectory is [14], [15] well-approximated by the single trajectory with the same initial conditions as the mean trajectory. The agreement between the single trajectory and mean trajectory breaks down when the position standard deviation saturates [14], [15], that is, when the position probability density becomes delocalized over the entire position interval. Figure 2 shows that the Newtonian and special-relativistic position standard deviations saturate at kick 19. Thus, in either the Newtonian (see Figure 3) or special-relativistic (see Figure 4) case, the mean trajectory is well-approximated by the single trajectory for the first 18 kicks only. The complete disagreement between the Newtonian and special-relativistic mean trajectories at kick 17 and kick 18 is therefore due to the complete disagreement of the Newtonian single trajectory and the special-relativistic single trajectory, which are both chaotic with Lyapunov exponent of 1.27, from kick 17 onwards. Since the position and momentum difference between the chaotic Newtonian and special-relativistic single trajectories grows exponentially at a rate approximately given by the Lyapunov exponent [4], we can estimate when the agreement between the two single trajectories breaks down and thus when the two mean trajectories breaks down. In particular, the position difference between the two single trajectories with the same initial conditions is 4.99×10^−9^ after 1 kick and, assuming that the exponential growth constant is 1.27, it grows to 0.1 (the maximum possible position difference is 1) after 14 kicks, which is close to the actual 17 kicks.

**Figure 5:**
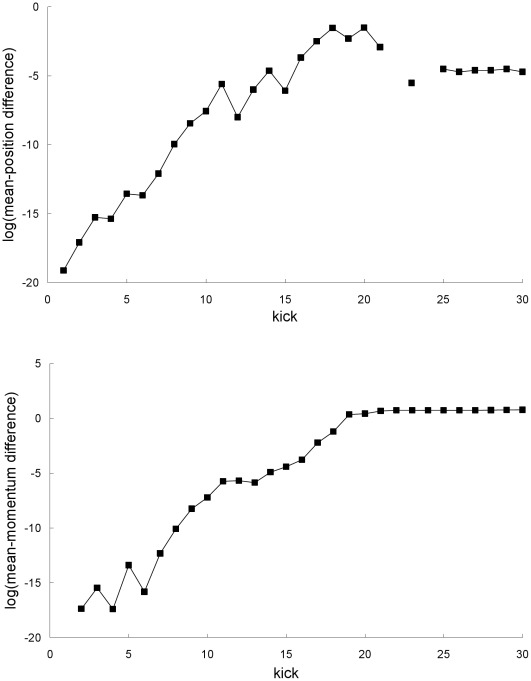
Difference between the mean trajectories for the first example. Natural-log of the absolute value of the difference between the Newtonian and special-relativistic mean positions (top plot) and mean momentums (bottom plot) for the first example. The mean-position differences at kick 22 and 24 cannot be resolved with the accuracy we have for the Newtonian and special-relativistic mean positions at those kicks.

Furthermore, the difference between the Newtonian and special-relativistic mean trajectories grow exponentially up to kick 18 (see Figure 5) because the difference between the Newtonian and special-relativistic chaotic single trajectories grow [4] exponentially. Hence, the breakdown of agreement between the Newtonian and special-relativistic mean trajectories is rapid because of the exponential growth of the difference between the two mean trajectories.

**Figure 6:**
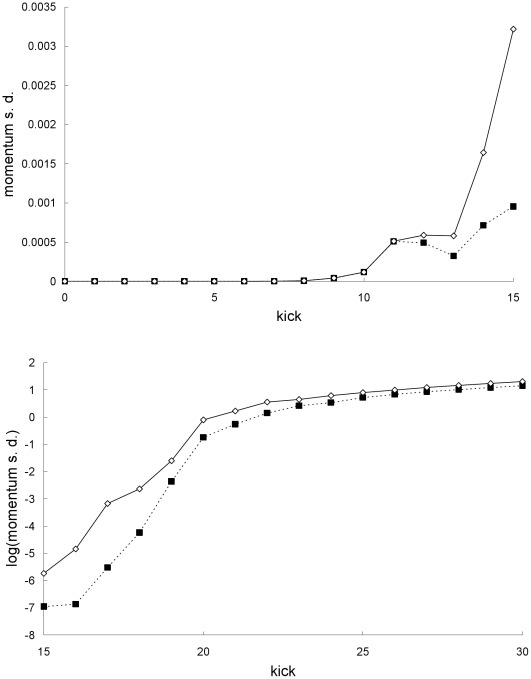
Comparison of momentum standard deviations for the first example. Newtonian (squares) and special-relativistic (diamonds) momentum standard deviations for the first example: first 15 kicks (top plot), kick 15 to 30 (bottom plot). The Newtonian and special-relativistic momentum standard deviations in the bottom plot are completely different from each other - they appear to be close from kick 25 onwards because the natural log of the standard deviations is plotted.

**Figure 7:**
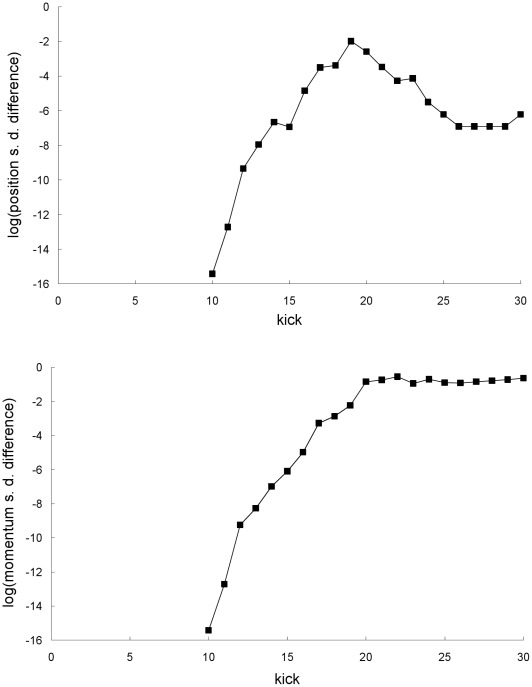
Difference between the standard deviations for the first example. Natural-log of the absolute value of the difference between the Newtonian and special-relativistic position standard deviations (top plot) and momentum standard deviations (bottom plot) for the first example. The standard-deviation differences from kick 1 to 9 cannot be resolved with the accuracy we have for the Newtonian and special-relativistic standard deviations at those kicks.

Figure 2 and Figure 6 show that the position and momentum standard deviations predicted by the two theories also do not always agree. The breakdown of agreement occurs at kick 12. This rapid breakdown of agreement is, see Figure 7, due to the exponential growth of the difference between the Newtonian and special-relativistic standard deviations, for both position and momentum, up to kick 12.

**Figure 8:**
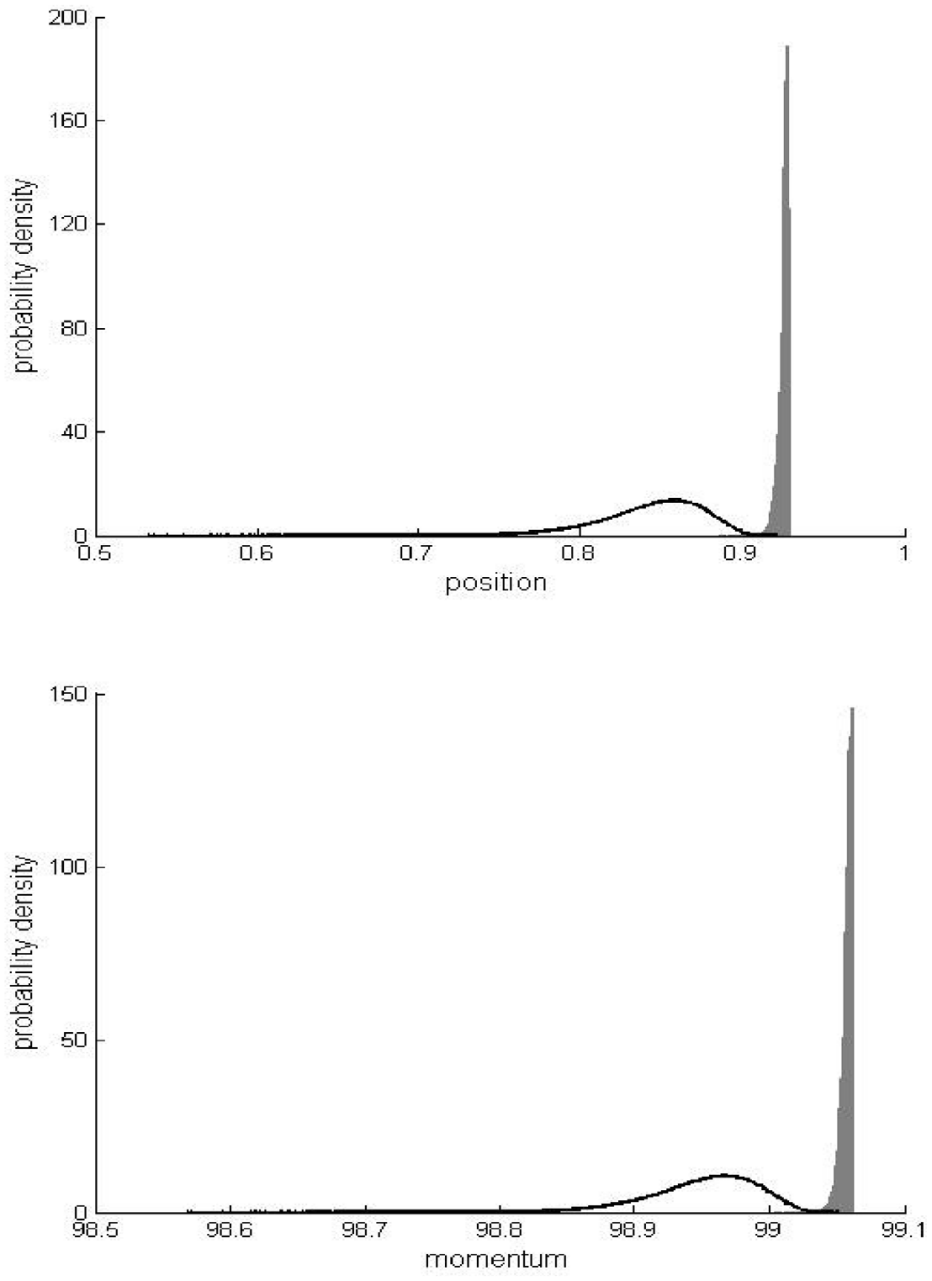
Comparison of probability densities for the first example. Newtonian (shaded grey) and special-relativistic (bold line) position (top plot) and momentum (bottom plot) probability densities for the first example at kick 17.

Together, Figure 1, Figure 2 and Figure 6 show that the agreement between the statistical predictions of the two theories, Newtonian and special relativistic, for the position and momentum means and standard deviations breaks down from kick 12 onwards even though the mean particle speed is low, only 0.001% of the speed of light since *β* = 10^−7^. Figure 8 shows the different Newtonian and special-relativistic position and momentum probability densities at kick 17.

**Figure 9:**
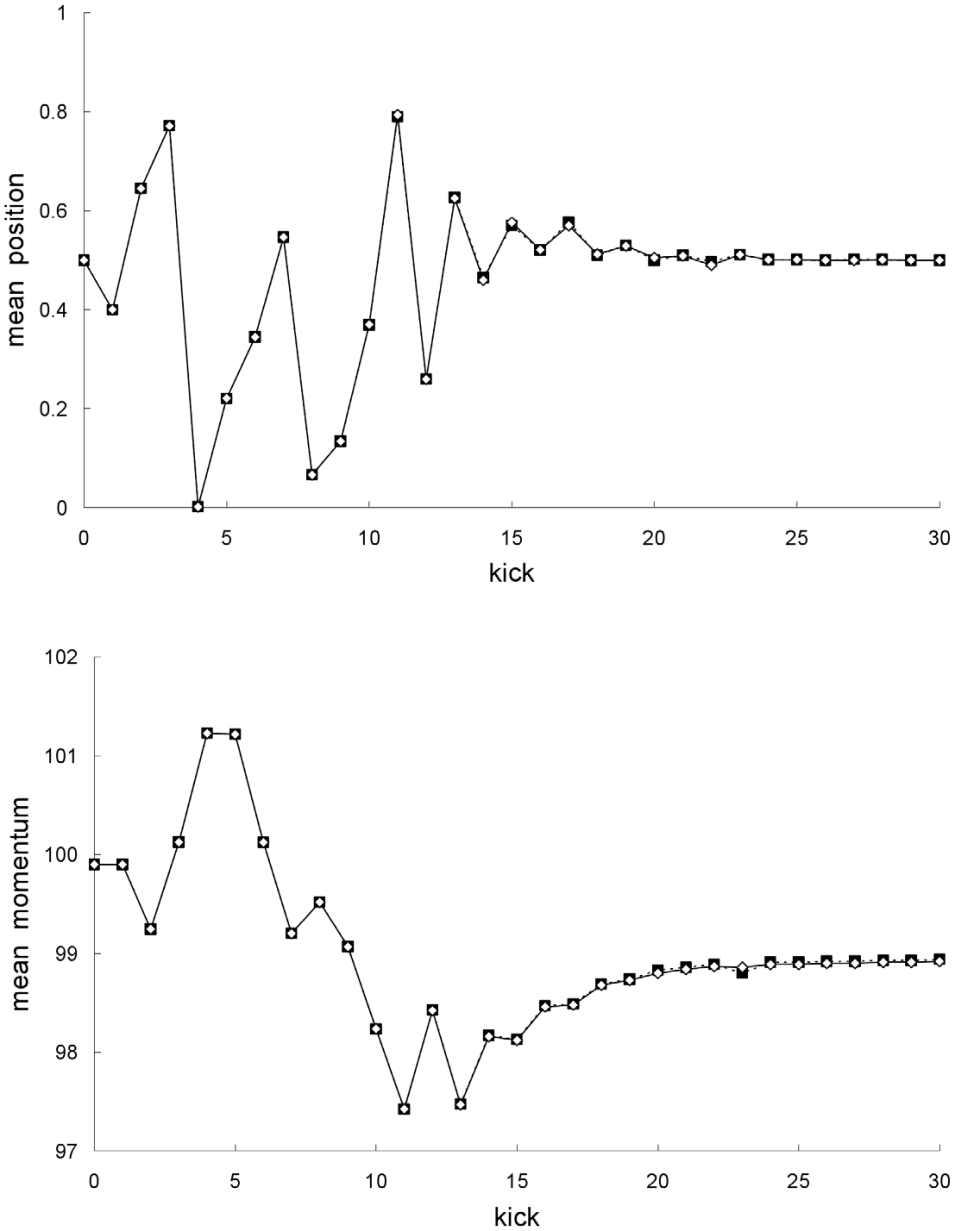
Comparison of mean trajectories for the second example. Newtonian (squares) and special-relativistic (diamonds) mean positions (top plot) and mean momentums (bottom plot) for the second example.

**Figure 10:**
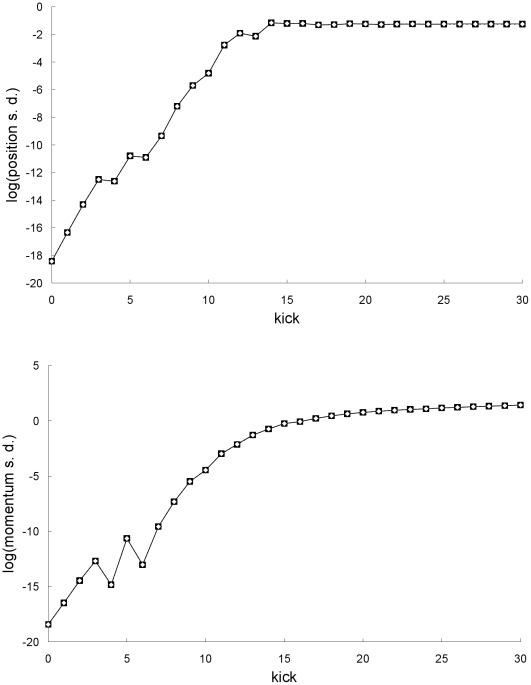
Comparison of standard deviations for the second example. Natural-log of the Newtonian (squares) and special-relativistic (diamonds) position standard deviations (top plot) and momentum standard deviations (bottom plot) for the second example.

In the second example, the parameters and initial means are the same as those in the first example but the initial standard deviations are larger: *σ_X0_* = *σ_P0_* = 10^−8^. In this case, Figure 9 shows there is no breakdown of agreement between the mean trajectory predictions of the two theories. In addition, Figure 10 shows there is also no breakdown of agreement between the position and momentum standard deviations predicted by the two theories.

The results in Figure 9 and Figure 10 for the second example can be understood as follows. Figure 3 and Figure 4 show that the single trajectory is close to the mean trajectory for the first 12 kicks only, in either the Newtonian or special-relativistic case. Thus, for the first 12 kicks, the Newtonian and special-relativistic mean trajectories are close because the Newtonian and special-relativistic single trajectories are close (recall, the agreement between the two single trajectories only breaks down at kick 17). Furthermore, the Newtonian and special-relativistic standard deviations are, like the means, still very close at kick 13 when the position standard deviations saturate. In other words, the Newtonian and special-relativistic position and momentum probability densities are essentially the same at kick 13. We have found that the agreement between the statistical predictions of the two theories for the position and momentum means and standard deviations does not break down for an ensemble of trajectories which is initially uniformly distributed (delocalized) in position. Thus, in this example, because the Newtonian and special-relativistic position probability densities are essentially the same and delocalized at kick 13, the statistical predictions of the two theories continue to be close for subsequent kicks.

The results illustrated by the two examples above were also found for other values of the *K* parameter: 0.9, 3.86, 6.4717, 6.9115 and 10.053.

Recall, in the first example (with smaller initial standard deviations), the Newtonian and special-relativistic position standard deviations saturate *after* the agreement between the Newtonian and special-relativistic single trajectories breaks down. However, in the second example (with larger initial standard deviations), the Newtonian and special-relativistic position standard deviations saturate *before* the agreement between the Newtonian and special-relativistic single trajectories breaks down. The first and second examples therefore show that in order for the statistical predictions of the two theories to break down, the initial Gaussian ensemble must be sufficiently well-localized in phase space, that is, the initial standard deviations must be sufficiently small such that the Newtonian and special-relativistic position standard deviations saturate *after* the agreement between the Newtonian and special-relativistic single trajectories breaks down.

If the initial ensemble is localized in the chaotic ‘sea’ in phase space, in the first example for instance, the agreement between the Newtonian and special-relativistic single trajectories (the initial conditions of the two single trajectories are the same, equal to the initial mean position and mean momentum) breaks down rapidly because the difference between the single trajectories grows [4] exponentially. In contrast, if the initial ensemble is localized in the non-chaotic ‘island’ in phase space, the difference between the Newtonian and special-relativistic single trajectories only grows [4] linearly, and therefore it takes a very long time for the agreement between the single trajectories to break down. This means that the breakdown of agreement between the statistical predictions of the two theories, Newtonian and special-relativistic, is very fast in the chaotic case, as we saw in the first example, but very slow in the non-chaotic case.

As an example of the non-chaotic case (this is our third example), for map parameters *K* = 0.9 and *β* = 10^−7^, the agreement between the Newtonian and special-relativistic single trajectories with initial conditions *X*_0_ = 0.7 and *P*_0_ = 99.9 takes about 10^8^ kicks [4] to break down. The Newtonian and special-relativistic statistical predictions can thus agree for a very long time. Indeed, for initial standard deviations *σ_X0_* = *σ_P0_* = 10^−9^, the means still agree to 6 and 9 significant figures respectively for position and momentum at kick 1000. At the same kick, the accuracies we have for both the Newtonian and special-relativistic standard deviations are 3 and 4 significant figures respectively for position and momentum – the Newtonian and special-relativistic standard deviations are the same, 2.67×10^−7^ for position and 2.446×10^−7^ for momentum, within these accuracies. Similar results were found for other non-chaotic cases for other values of the parameter *K*, 0.5 and 1.5.

Finally, the breakdown of agreement between the Newtonian and special-relativistic statistical predictions for the periodically-delta-kick particle at low speed can be further understood from a broader perspective by comparing the Newtonian Liouville’s equation for the phase-space probability density function *ρ*(*X*,*P*,*t*)
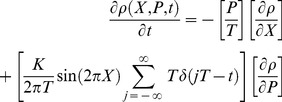
(7)with its special-relativistic counterpart
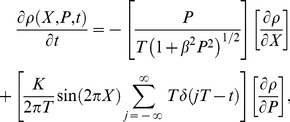
(8)where the infinite sum in both equations is the series of periodic delta kicks with period T. For low speed, βP<<1, therefore
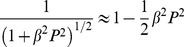
(9)in Eq. (8). The breakdown of agreement between the Newtonian and special-relativistic statistical predictions is therefore essentially due to the small β2P2/2 term in Eq. (8).

## Discussion

Since the periodically-delta-kicked particle is a prototypical [16] Hamiltonian system, we expect the breakdown of agreement between the Newtonian and special-relativistic statistical dynamical predictions to occur in other low-speed Hamiltonian systems.

Our finding raises an important fundamental question: When the Newtonian and special-relativistic statistical dynamical predictions are completely different for a low-speed system, which of the two predictions is empirically correct? Since special relativity continues to be successfully tested [17] in recent times, we expect the special-relativistic predictions to be correct. The breakdown of agreement therefore implies that special-relativistic mechanics must be used, instead of the standard practice of using Newtonian mechanics, to correctly calculate the statistical predictions for the dynamics of a low-speed system.

We have recently [18], [19] shown that the trajectory predicted by general-relativistic mechanics for a *low-speed weak-gravity* system is not always well-approximated by the trajectories predicted by special-relativistic and Newtonian mechanics for the same parameters and initial conditions. We expect similar breakdown of agreement in the statistical predictions for the mean, standard deviation and probability density function of the position and momentum. Finally, it would also be interesting to compare the thermodynamics predictions of classical Newtonian statistical mechanics with the predictions of classical special-relativistic statistical mechanics at low speed.
